# Identification of prognosis-related genes in the tumor microenvironment of stomach adenocarcinoma by TCGA and GEO datasets

**DOI:** 10.1042/BSR20200980

**Published:** 2020-10-13

**Authors:** Na Ren, Bin Liang, Yunhui Li

**Affiliations:** 1 Clinical Laboratory, General Hospital of Northern Theatre Command, Shenyang 110003, China; 2 Bioinformatics Department, Key laboratory of Cell Biology, Ministry of Public Health, and Key Laboratory of Medical Cell Biology, Ministry of Education, School of Life Sciences, China Medical University, China

**Keywords:** Bioinformatics, CIBERSORT, Differentially expressed genes, Stomach adenocarcinoma, Tumor microenvironment

## Abstract

Accumulating evidence has demonstrated that tumor microenvironment (TME) plays a crucial role in stomach adenocarcinoma (STAD) development, progression, prognosis and immunotherapeutic responses. How the genes in TME interact and behave is extremely crucial for tumor investigation. In the present study, we used gene expression data of STAD available from TCGA and GEO datasets to infer tumor purity using ESTIMATE algorithms, and predicted the associations between tumor purity and clinical features and clinical outcomes. Next, we calculated the differentially expressed genes (DEGs) from the comparisons of immune and stromal scores, and postulated key biological processes and pathways that the DEGs mainly involved in. Then, we analyzed the prognostic values of DEGs in TCGA dataset, and validated the results by GEO dataset. Finally, we used CIBERSORT computational algorithm to estimate the 22 tumor infiltrating immune cells (TIICs) subsets in STAD tissues. We found that stromal and immune scores were significantly correlated with STAD subtypes, clinical stages, *Helicobacter polyri* infection, and stromal scores could predict the clinical outcomes in STAD patients. Moreover, we screened 307 common DEGs in TCGA and GSE51105 datasets. In the prognosis analyses, we only found *OGN, JAM2, RERG, OLFML2B*, and *ADAMTS1* genes were significantly associated with overall survival in TCGA and GSE84437 datasets, and these genes were correlated with the fractions of T cells, B cells, macrophages, monocytes, NK cells and DC cells, respectively. Our comprehensive analyses for transcriptional data not only improved the understanding of characteristics of TME, but also provided the targets for individual therapy in STAD.

## Introduction

Stomach adenocarcinoma (STAD) is one of the most gastrointestinal malignancies, ranking the fifth in cancer incidence and the third in cancer mortality worldwide. Although the 5-year survival rate could reach 90–97% at early diagnosis and early treatment, the 5-year survival rate is less than 30% in advanced stage or metastatic disease in STAD patients [1,2]. STAD, as a well-recognized heterogeneous cancer, is influenced by a range of environmental and genetic factors. The malignant phenotypes of STAD are defined not only by the intrinsic activities of cancer cells but also by the stromal and immune cells recruited and activated in the tumor-related microenvironment [3]. Accumulating evidence has demonstrated a crucial role for the tumor microenvironment (TME) in STAD development, progression, prognosis and immunotherapeutic responses [4,5]. Therefore, elucidating the characteristics of TME in STAD would enlarge our understanding of pathological mechanism in STAD and provide a basis for further individual therapy.

A solid tumor consists of various cell components, including cancer cells, stromal cells, fibroblasts, smooth muscle cells, endothelial cells, and hematopoietic cells, macrophages and lymphocytes. Among them, immune cells and stromal cells are the major components necessary for tumor development and progression. How the components in TME interact and behave is extremely crucial for tumor investigation. Current studies have indicated that the function of stromal cells and interaction with tumor cells contribute to the tumor progression, tumor invasion, and tumor spread [6]. Moreover, the stromal cells could secret growth factors, cytokines, and chemokines, which have substantial impact on tumor characteristics [7]. STAD is *Helicobacter pylori*-induced chronic gastritis, and frequently characterized by the immune cell infiltration, containing granulocytes, macrophages, and T lymphocytes [8,9]. Tumor-associated lymphocytes (TALs), primarily T cells, are the major type of infiltrating immune cells in cancer tissues, and produce soluble cytokines that regulate the proliferation and migration of cancer cells, promote angiogenesis, and participate in activation of host defense mechanism [10–12]. In STAD, tumor-associated macrophages (TAMs) infiltration into tumor tissue was correlates significantly with tumor vascularity, the depth of tumor invasion, lymph node status and clinical stages [13,14]. Furthermore, alterations in the TME may facilitate the development of STAD heterogeneity through the extrinsic activation of various genes and signaling pathways. Targeting the key molecules and interrupting the key signaling interactions in the TME may be a promising therapeutic strategy for STAD patients.

In the present study, we used gene expression data of STAD available from TCGA and GEO datasets to infer tumor purity using Estimation of STromal and Immune cells in MAlignant Tumor tissues using Expression data (ESTIMATE) algorithms, and predicted the associations between stromal cells/immune cells and clinical features and clinical outcomes. Then, we screened the differentially expressed genes (DEGs) from the comparisons of immune and stromal scores, and postulated key biological processes and pathways that DEGs mainly involved in. From the overlapping DEGs in TCGA and GEO datasets, we evaluated the prognostic values of common genes in TCGA and GEO datasets. Finally, The Cell-type Identification By Estimating Relative Subsets Of RNA Transcript (CIBERSORT), a newly proposed computational algorithm, was conducted to estimate the fractions of 22 tumor infiltrating immune cells (TIICs) subsets in STAD tissues, and compared the differences in different prognosis-related gene expression levels. Therefore, we aimed to provide reliable information that would be effective in predicting the prognosis, monitoring the alteration of TME, and guiding individual treatment in STAD.

## Materials and methods

### Raw data

The RNA sequencing data and corresponding clinical information of STAD patients were obtained from TCGA data portal (https://tcga-data.nci.nih.gov/tcga/). The inclusion criteria of STAD samples were as follows: (i) gene expression profiling of STAD were available in the dataset; (ii) complete clinical data of STAD patients were required, including gender, age, TNM stage, and overall survival. Finally, a total of 408 STAD patients were enrolled in the study. We performed systematic search in the GEO database (https://www.ncbi.nlm.nih.gov/geo/) to find the gastric cancer gene expression datasets. Two independent datasets from the GEO database were used for external validation in this study, including GSE51105 and GSE84437 datasets. The GSE51105 included 94 GC samples, and GSE84437 included 433 GC samples with available clinical information.

### Estimation of stromal and immune scores

The stromal scores and immune scores in TCGA STAD dataset and GSE51105 dataset were calculated using ESTIMATE algorithm (https://bioinformatics.mdanderson.org/estimate/) to estimate the tumor purity for each STAD sample. Then, STAD patients in TCGA dataset were divided into two groups according to the median value of stromal score (high stromal score group and low stromal score group) and immune score (high immune score group and low immune score group). For TCGA STAD samples, the correlations between stromal/immune scores and clinical features were analyzed. The prognostic values of stromal score and immune score were evaluated by Kaplan–Meier method, and examined by Log-rank test.

### Identification of DEGs in TCGA and GSE51105 datasets

The samples of TCGA dataset and GSE51105 dataset were divided into two groups according stromal scores (high vs. low) and immune scores (high vs. low), respectively. DEGs were identified using *limma* package of R software. DEGs between high score and low score need to satisfy the following criterions: log_2_ | fold change (FC) | ≥ 0.5 and *P*-value < 0.05. The overlapping genes in TCGA dataset and GSE51105 were identified between stromal group and immune group, respectively.

### Functional enrichment analysis of DEGs

To further explore the common characteristics, the overlapping DEGs were identified using Venn diagram. Then, the overlapping DEGs were analyzed by the Database for Annotation, Visualization and Integrated Discovery (DAVID) bioinformatics tool (https://david.ncifcrf.gov/). Gene Ontology (GO) and Kyoto Encyclopedia of Genes and Genomes (KEGG) pathway enrichment analyses were then performed to annotate the potential functions for overlapping DEGs. *P*-value <0.05 was considered to be significant.

### Protein–protein interaction analysis

The interactive relationships among common DEGs were analyzed by constructing a protein–protein interaction (PPI) network using The Search Tool for the Retrieval of Interacting Genes database (STRING, www.string-db.org/). An interaction score >0.9 was set as cut-off value.

### Prognosis-related genes screening and validation

Kaplan–Meier plots of common genes in TCGA dataset and GSE51105 dataset were constructed to analyze the overall survival (OS), and examined by Log-Rank test. For result robustness, GSE84437 dataset was used as a validation group. *P*<0.05 was considered statistically to be significant.

### Association between prognosis-related genes and immune cell type fractions

Normalized gene expression data of TCGA STAD dataset were used to infer the relative proportions of 22 TIIC subtypes using the CIBERSORT algorithm (https://cibersort.stanford.edu/). The default reference leukocyte gene signature (LM22) is a matrix file consisting of 547 genes that accurately distinguish 22 mature human hematopoietic populations isolated from peripheral blood or *in vitro* culture conditions, including seven T cell types, naive and memory B cells, plasma cells, natural killer (NK) cells, and myeloid subsets [15]. Only patients with a CIBERSORT *P *< 0.05 were considered to be eligible for further analyses. For each sample, the sum of all estimates of immune cell type fractions equaled to 1. Comparisons of proportions of TIICs were performed between low and high expression groups based on prognosis-related gene expression in STAD patients. *P*<0.05 was considered statistically significant.

## Results

### Correlation of stromal score and immune score in different clinical characteristics in STAD patients

**Figure 1:**
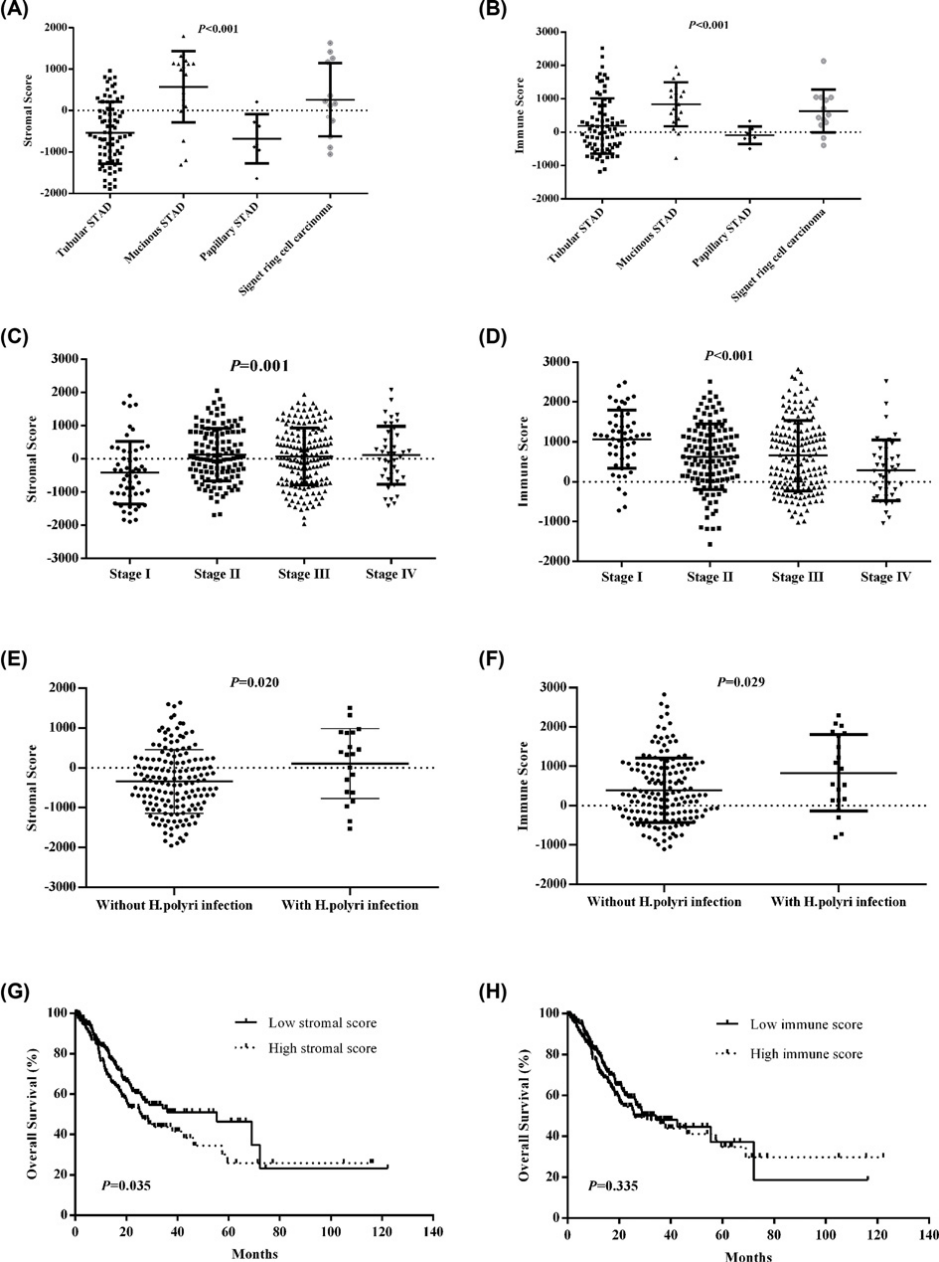
The comparisons of stromal scores and immune scores in different clinical characteristics and clinical outcomes (**A** and **B**) The differences of stromal scores and immune scores in varied cancer types. (**C** and **D**) The differences of stromal scores and immune scores in clinical stages. (**E** and **F**) The differences of stromal scores and immune scores between without *H. polyri* infection and with *H. polyri* infection. (**G** and **H**) Associations of stromal scores and immune scores with overall survival.

The gene expression and clinical information were downloaded from TCGA database. Among them, 262 patients (64.2%) were males, and 146 patients (35.8%) were females. The mean age of all patients were 65.6 ± 10.6 years (range: 30–90 years). Of 408 patients, 76 patients were tubular stomach adenocarcinoma, 20 patients were mucinous stomach adenocarcinoma, 7 patients were papillary stomach adenocarcinoma, and 13 patients were signet ring cell carcinoma, 223 patients were stomach adenocarcinoma (without pathological type), and 69 patients were diffuse type stomach adenocarcinoma. In the clinical stage classification, 52 patients (13.6%) were in stage I, 122 patients (31.9%) were in stage II, 167 patients (43.7%) were in stage III, and 41 patients (10.7%) were in stage IV. Distant metastasis happened in 27 patients (6.9%). Then, we examined the distribution of stromal and immune scores in each STAD patient using ESTIMATE algorithm. The stromal scores ranged from -1957.19 to 2085.81, and immune scores ranged from -1568.74 to 2826.73. We found that stromal scores and immune scores were significantly different in varied cancer types (all *P*<0.001, Figure 1A,B). According to stromal scores, mucinous type ranked the highest, followed by signet ring type and tubular type, and papillary type was the lowest. Otherwise, the rank order of immune score in STAD patients was mucinous type, signet ring type, tubular type, and papillary type. Base on the clinical stage classification, the stromal scores and immune scores were significantly different in different stages (stromal score: *P*=0.001; immune score: *P*<0.001, Figure 1C,D). Moreover, we found that stromal scores and immune scores in STAD patients with *helicobacter pylori (H. polyri)* infection were significantly higher than STAD patients without *H. polyri* infection (stromal score: *P*=0.020; immune score: *P*=0.029, Figure 1E,F). In survival analyses, the patients with low stromal score had longer OS than the patients with high stromal score (OS: *P*=0.035, Figure 1G). No significant difference was found in OS between low immune score group and high immune score group (*P*=0.335, Figure 1H).

### Identification of DEGs between different stromal/immune score groups

**Figure 2:**
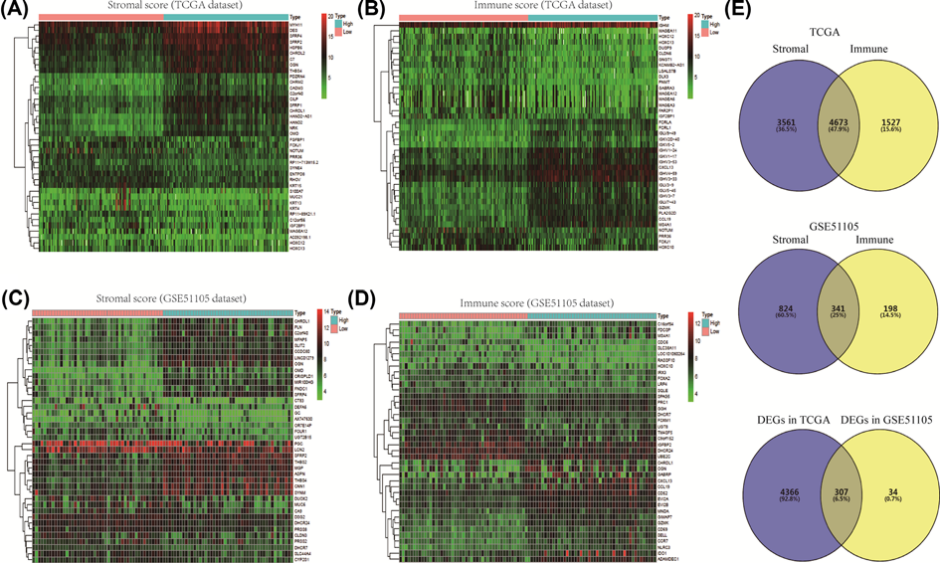
Differentially expressed genes (DEGs) between low versus high (stromal score/immune score) (**A**) Heatmap of top 50 DEGs of stromal scores (low score vs. high score) in TCGA dataset. (**B**) Heatmap of top 50 DEGs of immune scores (low score vs. high score) in TCGA dataset. (**C**) Heatmap of top 50 DEGs of stromal scores (low score vs. high score) in GSE51105 dataset. (**D**) Heatmap of top 50 DEGs of immune scores (low score vs. high score) in GSE51105 dataset. (**E**) Overlapping genes between TCGA DEGs and GSE51105 DEGs.

We screened the DEGs between low stromal score and high stromal score samples, and low immune score and high immune score samples in TCGA dataset and GSE51105 dataset, respectively. According to different stromal score and immune score, we plotted the heatmaps of top 50 DEGs in TCGA dataset and GSE51105 dataset, respectively (Figure 2A–D). A total of 8,234 DEGs, including 5,091 up-regulated genes and 3,143 down-regulated genes, were screened according to stromal scores in TCGA dataset. In addition, there were 3,943 up-regulated genes and 2,257 down-regulated genes between low immune score and high immune score samples in TCGA dataset. The overlapping gene number between stromal group and immune group was 4,673. Moreover, the number overlapping genes in GSE51105 was 341. Finally, we screened 307 common genes between TCGA DEGs and GSE51105 DEGs for result reliability (Figure 2D).

### GO function and KEGG pathway analyses

**Figure 3:**
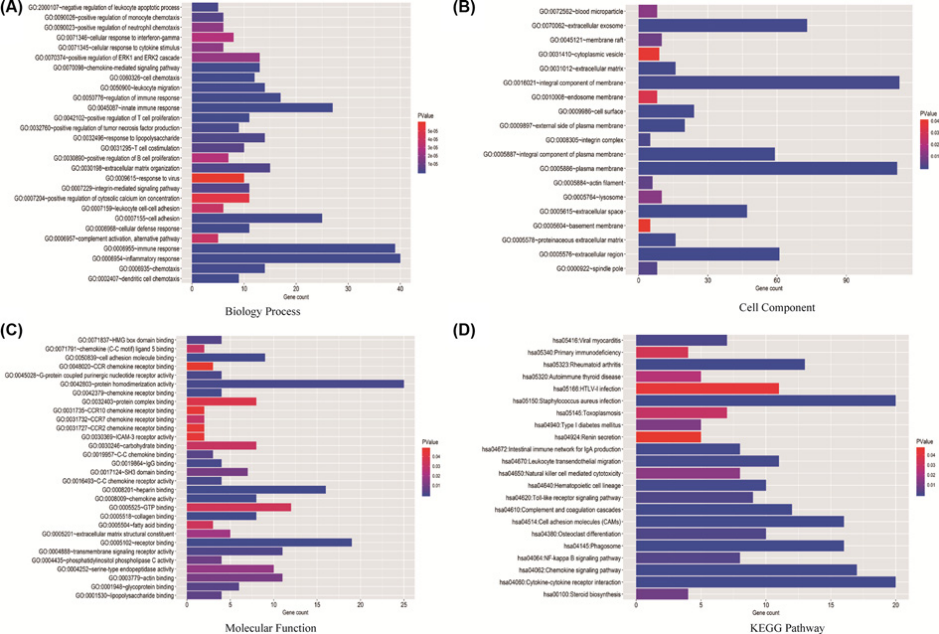
The GO and KEGG function enrichment analyses of overlapping DEGs (**A**) The GO term, biological processes of 307 common DEGs. (**B**) The GO term, cell components of 307 common DEGs. (**C**) The GO term, molecular functions of 307 common DEGs. (**D**) KEGG pathway enrichment analyses of 307 common DEGs.

Then, GO function and KEGG pathway enrichment analyses were performed for common DEGs. The common DEGs were enriched in biological processes, such as inflammatory response, immune response, dendritic cell chemotaxis, chemokine-mediated signaling pathway, cell chemotaxis, positive regulation of T cell proliferation, cellular defense response, and leukocyte migration (Figure 3A). These common DEGs were mainly located in integral component of membrane, plasma membrane, and extracellular exosome (Figure 3B). From the molecular function, it was observed that the common DEGs were enriched in heparin binding, cell adhesion molecule binding, receptor binding, chemokine activity, and collagen binding (Figure 3C). The KEGG pathways analyses showed that the DEGs were mainly concentrated in cytokine-cytokine receptor interaction, chemokine signaling pathway, cell adhesion molecules, phagosome, complement and coagulation cascades, leukocyte transendothelial migration, hematopoietic cell lineage, and Toll-like receptor signaling pathway (Figure 3D).

### PPI network construction

**Figure 4:**
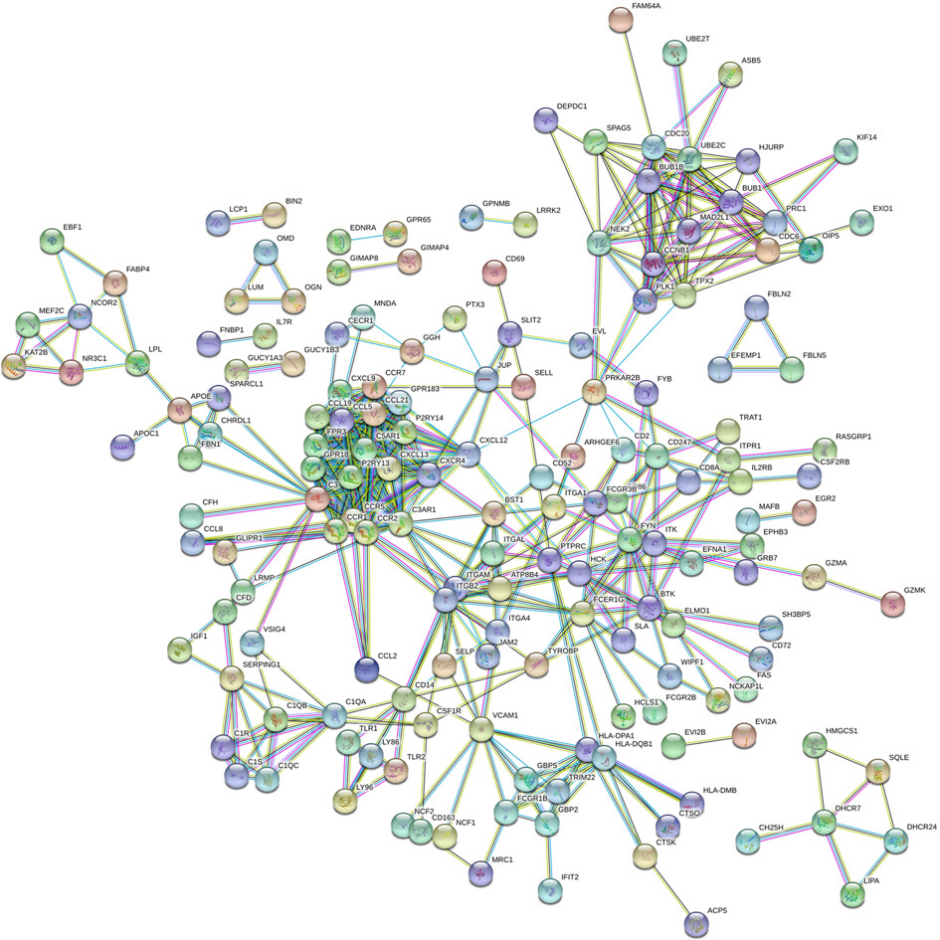
PPI network of common DEGs in STRING database

The common DEGs were analyzed using the online STRING database under the cut-off: interaction score > 0.9, revealing 300 nodes and 793 edges. The average node degree was 5.29. The interactions of common DEGs were shown in Figure 4.

### Survival analyses of common DEGs

**Figure 5:**
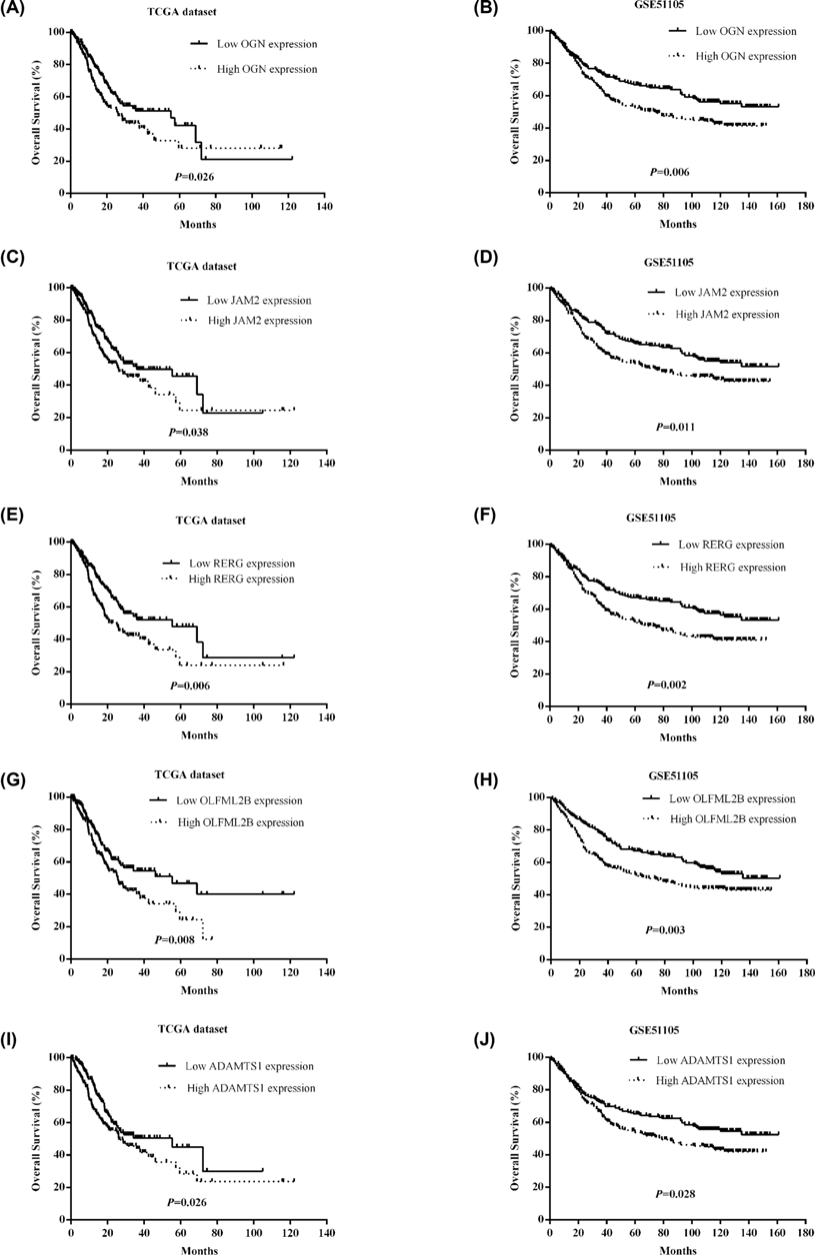
Prognosis-related genes in STAD patients in TCGA and GSE84437 datasets (**A**) OGN in TCGA dataset; (**B**) OGN in GSE84437 dataset; (**C**) JAM2 in TCGA dataset; (**D**) JAM2 in GSE84437 dataset; (**E**) RERG in TCGA dataset; (**F**) RERG in GSE84437 dataset; (**G**) OLFML2B in TCGA dataset; (**H**) OLFML2B in GSE84437 dataset; (**I**) ADAMTS1 in TCGA dataset; (**J**) ADAMTS1 in GSE84437 dataset.

The OS were evaluated by Kaplan–Meier method for 307 common DEGs in TCGA dataset. For result robustness, GSE84437 dataset was selected as validation group. The results indicated that osteoglycin (*OGN*) (TCGA: *P*=0.026; GSE84437: *P*=0.006), junctional adhesion molecule 2 (*JAM2*) (TCGA: *P*=0.038; GSE84437: *P*=0.011), Ras-like estrogen-regulated growth inhibitor (*RERG*) (TCGA: *P*=0.006; GSE84437: *P*=0.002), olfactomedin-likes 2B (*OFLML2B*) (TCGA: *P*=0.008; GSE84437: *P*=0.003), and thrombospondin motif type 1 (*ADAMTS1*) (TCGA: *P*=0.026; GSE84437: *P*=0.028) were significantly associated with OS, not only in TCGA group, but also in GSE84437 group (Figure 5).

### Correlation of prognosis-related genes with TIICs in STAD patients

**Figure 6:**
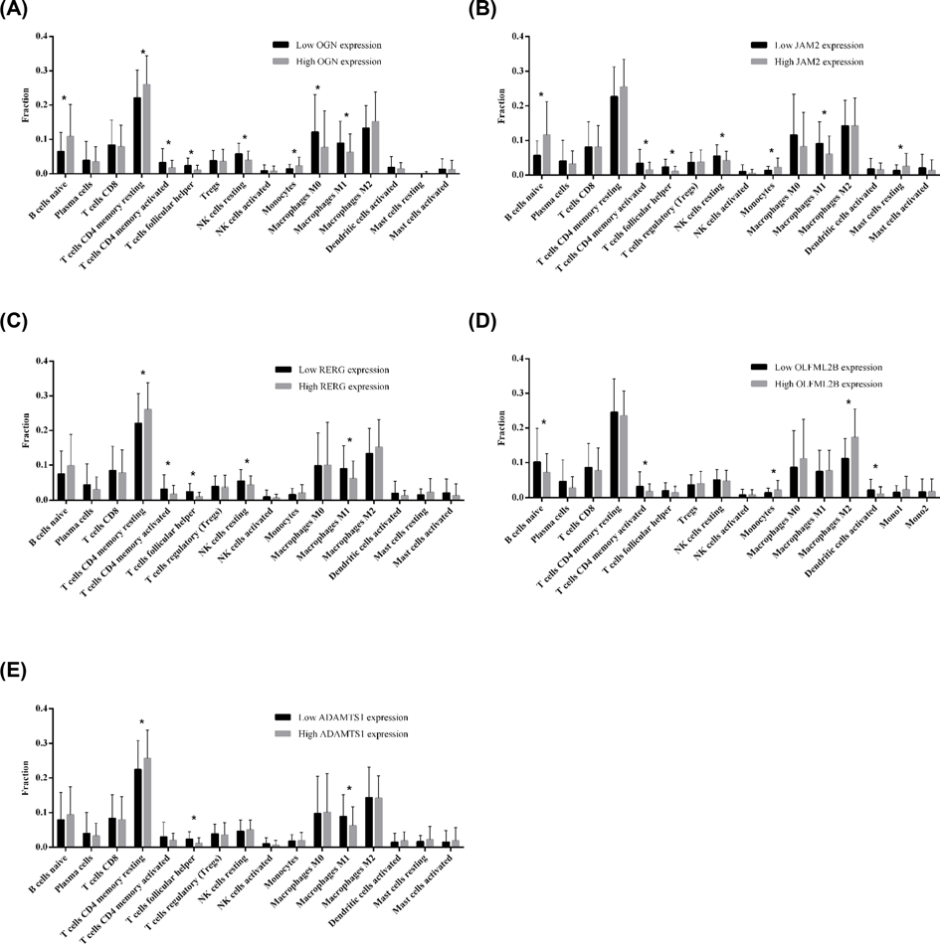
Differences of TIICs between low expression and high expression of OGN, JAM2, RERG, OLFML2B and ADAMTS1 in STAD patients **(A)** OGN; (**B**) JAM2; (**C**) RERG; (**D**) OLFML2B; (**E**) ADAMTS1. * *P*<0.05.

We analyzed the correlation between *OGN, JAM2, RERG, OFLML2B*, and *ADAMTS1* levels and TIICs. The result indicated that the fractions of naïve B cells, resting CD4^+^ memory T cells, activated CD4^+^ memory T cells, follicular helper T cells (Tfh cells), macrophage M0 and M1, resting NK cells and monocytes were significant different between low *OGN* expression and high *OGN* expression groups (all *P*<0.05, Figure 6A). *JAM2* expression was significantly correlated with the fractions of naïve B cells, activated CD4^+^ memory T cells, Tfh cells, macrophage M1, resting mast cells, resting NK cells and monocytes (all *P*<0.05, Figure 6B). The fractions of resting CD4^+^ memory T cells, activated CD4^+^ memory T cells, Tfh cells, macrophage M1, and resting NK cells were significantly different between low *RERG* expression and high *RERG* expression groups (all *P*<0.05, Figure 6C). Moreover, the fractions of naïve B cells, activated CD4^+^ memory T cells, macrophages M2, activated DC cells, and monocytes were significantly different between low *OFLML2B* expression and high *OFLML2B* expression groups (all *P*<0.05, Figure 6D). Finally, *ADAMTS1* expression was only significantly associated with resting CD4^+^ memory T cells, Tfh cells and macrophages M2 (all *P*<0.05, Figure 6E).

## Discussion

TME mainly participates in maintaining cell homeostasis in the normal tissues, but TME in the tumor is significantly different with healthy tissue in cell fraction and cell status [16]. A growing body of literature suggested a key role for the TME in the pathogenesis of gastric cancer, which indicated the biological behaviors of gastric cancer were correlated to not only cancer cells but also TME. As mentioned above, the TME in STAD is composed of cancer cells and non-cancer cells, mainly including stromal cells and immune cells [17]. Stromal cell could promote tumor growth and influence the cancer behaviors, whereas immune infiltrating cells could inhibit tumor progression [18,19]. ESTIMATE, as a new algorithm, adopt transcriptional data of cancer samples to calculate the tumor purity according to stromal scores and immune scores. Stromal scores and immune scores were inferred through the infiltration level of stromal cells and immune cells (nontumor cells) in tumor tissues using gene expression data. Our study suggested that stromal scores and immune scores were significantly different in varied cancer types, clinical stages and *H. polyri* infection. Moreover, the high stromal scores indicated worse clinical outcomes. Therefore, we speculated that the infiltration of immune cells and stromal cells in TME was different in each tumor samples, implying that some genes were differentially expressed in tumor tissues.

According to gene expression data, we analyzed the DEGs between high stromal score and low stromal score groups (or high immune score and low immune score groups), and screened the overlapping genes between stromal cell and immune cell groups. For the credibility of screening, we used GSE51105 dataset as a validation group. A total of 307 common DEGs were identified. The biological functions of common DEGs were mainly in inflammatory response, immune response, dendritic cell chemotaxis, chemokine-mediated signaling pathway, cell chemotaxis, positive regulation of T-cell proliferation, cellular defense response, and leukocyte migration. Moreover, the common DEGs were involved in a series of immune and inflammatory related signal pathways, such as cytokine–cytokine receptor interaction, chemokine signaling pathway, cell adhesion molecules, complement and coagulation cascades, leukocyte transendothelial migration, and Toll-like receptor (TLR) signaling pathway. In addition, the TLR signaling pathway are classical pattern recognition receptor pathway in the recognition of *H. pylori* and medicates the host immune responses against *H. pylori* [20]*.* Recent study indicated that up-regulated TLR2 was associated with advanced stage, distant metastasis, and poor clinical outcomes in gastric cancer [21]. Therefore, focusing on these biological function and pathways will help us elucidate the underlying mechanisms of STAD. Furthermore, the enrichment analyses also indicated that the immune and inflammatory signal pathways were closely related the development and progression of STAD.

Next, we analyzed the prognostic values of 307 common DEGs in TCGA dataset, and validated the genes through GSE84437 dataset. We found that five genes, *OGN, JAM2, RERG, OFLML2B* and *ADAMTS1*, were significantly correlated with overall survival. OGN, a small leucine-rich proteoglycan family member, was found down-regulated in cervical cancer [22], gastric cancer [23], and colorectal cancer [24]. Xu et al*.* reported that OGN inhibited cell proliferation, migration, and invasiveness in breast cancer MCF-7 cells via PI3K/Akt/mTOR signaling pathway [25]. OGN could reverse epithelial-to-mesenchymal transition (EMT) and inhibit invasiveness in colon cancer cells [26]. JAM2 belongs to an immunoglobulin subfamily, and could regulate tight junction assembly and leukocyte recruitment during inflammation, angiogenesis, cell proliferation, and migration [27]. Zhao et al. demonstrated that decreased JAM-2 expression was significantly associated with cancer progression, metastasis, and poor prognosis in colorectal cancer [28]. More studies suggested that JAM2 expression was different in various types of cancers. The inconsistent results leaded to the uncertainty of JAM-2 function in tumorogenesis. *RERG* located on chromosome 12p12, encoding a small GTP-binding and hydrolyzing protein (GTPase) of the Ras superfamily [29]. *RERG* was initially identified as a tumor suppressor gene in breast cancer, and RERG overexpression showed an association with better prognosis in ER-positive luminal-like subtype [29,30]. Zhao et al*.* found that RERG inhibited tumor growth and angiogenesis by down-regulating MMP2, MMP9, IL-6, and IL-8, and suppressed ERK/NF-κB signaling pathway in nasopharyngeal carcinoma xenografts in nude mice [31]. But, the potential ability and detailed mechanism of RERG in gastric cancer requires further study. *OLFML2B*, located on chromosome 1q23.3, was first identified and characterized in mouse retina [32,33]. In gastric cancer, OLFML2B could promote the growth of gastric cancer cells, maybe serving an oncogene in the development of gastric cancer [34]. However, the molecular mechanism of OLFML2B remains unclear in tumorogenesis. ADAMTS1, belonging to the zinc-dependent metzincin superfamily, was originally identified in cachexigenic colon cancer cells [35,36]. Accumulating evidence has demonstrated that ADAMTS1 has metastasis potential by degrading the ECM and regulating growth factors and adhesion molecules [37]. ADAMTS1 expression was dysregulated in primary gastric cancer and paired lymph node metastasis [38]. Recent study found that ADAMTS1 was required for a balanced immune cell repertoire and tumor inflammatory response via affecting lymphocyte and myeloid populations in the spleen and bone marrow [39]. In the present study, we analyzed the correlations between *OGN, JAM2, RERG, OLFML2B*, and *ADAMTS1* expressions and clinical characteristics (clinical stage, grade, N stage, T stage, and *H. polyri* infection) (Supplementary Figure S1). We found that *OGN, JAM2, RERG, OLFML2B* and *ADAMTS1* expressions at mRNA levels were significantly associated with different clinical characteristics, respectively. Therefore, further biological experiments *in vivo* and *in vitro* are required to confirm the functions of *OGN, JAM2, RERG, OLFML2B* and *ADAMTS1* genes in gastric cancer in the future.

CIBERSORT is an algorithm that allows for highly sensitive and specific discrimination of 22 human immune cell phenotypes, with applications for identifying cellular biomarkers and novel therapeutic targets [40]. We compared the fractions of TIICs in high and low *OGN, JAM2, RERG, OLFML2B* and *ADAMTS1* expression groups. We found that their expressions were significantly correlated with T cells, B cells, macrophages, monocytes, NK cells, and DC cells, respectively. At present, immunotherapy has always been a potential part of comprehensive therapy for gastric cancer through blocking immune checkpoints, such as cytotoxic T-lymphocyte-associated antigen 4 (CTLA-4), programmed cell death-1 (PD-1), and its ligand (PD-L1) [41]. However, there is still a large proportion of patients exhibiting limitation in immunotherapy due to TME heterogeneity, immunosupression and off-target effect in cancer patients [42,43]. The accumulating literatures suggested that the efficacy of immunotherapy seems to be associated with TME [44], and TIICs could be prognostic biomarkers and differently influence the clinical outcomes in gastric cancer patients [45]. Therefore, understanding the tumor immune infiltration and immune related gene expression is helpful for improving immunotherapy approaches.

## Conclusions

In the present study, we identified *OGN, JAM2, RERG, OFLML2B*, and *ADAMTS1* were significantly correlated with clinical outcome using TCGA and GEO datasets, and illuminate the associations between prognosis-related genes and TIICs in TME. Our comprehensive analyses for transcriptional data not only improved the understanding of characteristics of TME, but also provided the targets for individual therapy in STAD. Further research is needed to find the underlying molecular mechanisms and validate our analyses results.

## Supplementary Material

Supplementary Figure S1

## Abbreviations

CTLA-4: cytotoxic T-lymphocyte-associated antigen 4
DEG: differentially expressed gene
PD-1: programmed cell death-1
STAD: stomach adenocarcinoma
TLR: toll-like receptor
TME: tumor microenvironment
