# Supplementary material for: Identification of prognosis-related genes in the tumor microenvironment of stomach adenocarcinoma by TCGA and GEO datasets
Source: Biosci Rep. 2020 Oct 13;40(10):BSR20200980. doi: 10.1042/BSR20200980 (PMC7560520; doi:10.1042/BSR20200980)
Supplement: Supplementary Figure S1 [file BSR-2020-0980_supp.pdf]

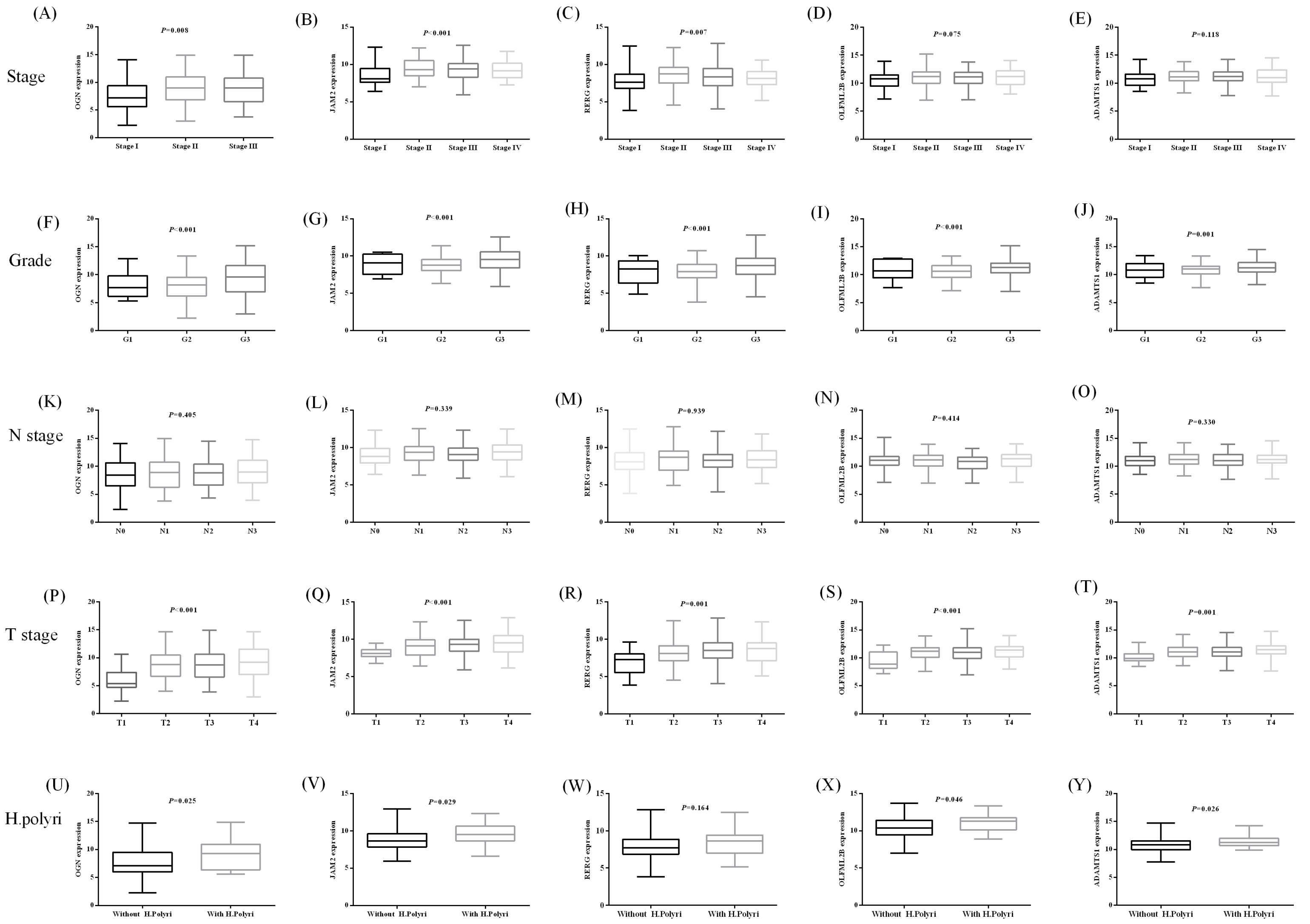

Stage
Grade
N stage
T stage
H.polyri
(A) P=0.008
(B) P<0.001
(C) P=0.007
(D) P=0.075
(E) P=0.118
(F) P<0.001
(G) P<0.001
(H) P<0.001
(I) P<0.001
(J) P=0.001
(K) P=0.405
(L) P=0.339
(M) P=0.939
(N) P=0.414
(O) P=0.330
(P) P<0.001
(Q) P<0.001
(R) P=0.001
(S) P<0.001
(T) P=0.001
(U) P=0.025
(V) P=0.029
(W) P=0.164
(X) P=0.046
(Y) P=0.026
OGN expression
JAM2 expression
RERG expression
OLFML2B expression
ADAMTS1 expression
Stage I
Stage II
Stage III
Stage IV
G1
G2
G3
N0
N1
N2
N3
T1
T2
T3
T4
Without H.Polyri
With H.Polyri

Figure S1. Correlation between OGN, JAM2, RERG, OLFML2B and ADAMTS1 expression and clinical features. (A-E) Clinical stage; (F-J) Grade; (K-O) N stage; (P-T) T stage; (U-Y) *H.polyri* infection
